# The three-tails approach as a new strategy to improve selectivity of action of sulphonamide inhibitors against tumour-associated carbonic anhydrase IX and XII

**DOI:** 10.1080/14756366.2022.2053526

**Published:** 2022-03-21

**Authors:** Alessandro Bonardi, Silvia Bua, Jacob Combs, Carrie Lomelino, Jacob Andring, Sameh Mohamed Osman, Alessandra Toti, Lorenzo Di Cesare Mannelli, Paola Gratteri, Carla Ghelardini, Robert McKenna, Alessio Nocentini, Claudiu T. Supuran

**Affiliations:** a Department NEUROFARBA – Pharmaceutical and Nutraceutical Section, University of Firenze, Florence, Italy; b Department NEUROFARBA – Pharmaceutical and Nutraceutical Section, Laboratory of Molecular Modeling Cheminformatics & QSAR, University of Firenze, Florence, Italy; c Department of Biochemistry and Molecular Biology, College of Medicine, University of Florida, Gainesville, FL, USA; d Chemistry Department, College of Science, King Saud University, Riyadh, Saudi Arabia

**Keywords:** Tail approach, carbonic anhydrase, inhibitor, X-ray crystallography, hypoxic tumour

## Abstract

Human (h) carbonic anhydrase (CAs, EC 4.2.1.1) isoforms IX and XII were recently confirmed as anticancer targets against solid hypoxic tumours. The “three-tails approach” has been proposed as an extension of the forerunner “tail” and “dual-tail approach” to fully exploit the amino acid differences at the medium/outer active site rims among different hCAs and to obtain more isoform-selective inhibitors. Many three-tailed inhibitors (TTIs) showed higher selectivity against the tumour-associated isoforms hCA IX and XII with respect to the off-targets hCA I and II. X-ray crystallography studies were performed to investigate the binding mode of four TTIs in complex with a hCA IX mimic. The ability of the most potent and selective TTIs to reduce *in vitro* the viability of colon cancer (HT29), prostate adenocarcinoma (PC3), and breast cancer (ZR75-1) cell lines was evaluated in normoxic (21% O_2_) and hypoxic (3% O_2_) conditions demonstrating relevant anti-proliferative effects.

## 1. Introduction

Tumour growth, malignant progression, and resistance to chemotherapy and radiotherapy appear to be strongly associated with tumour hypoxia^1–5^. Hypoxia is the main cause responsible for the overexpression of the hypoxia-inducible factor (HIF-1) and the Warburg effect in tumours, an indispensable metabolic reprogramming of cancer cells from glycolytic metabolism to fermentation^1–8^. In order to survive in hypoxic conditions and acidosis due to fermentative metabolism, HIF-1 triggers a signalling cascade, that upregulates the expression of several genes, coding for the lactate-proton symporters (MCT4), other proton-transporters, and the tumour-associated isoforms IX and XII of carbonic anhydrases (CAs, EC 4.2.1.1), that catalyse the reversible hydration of carbon dioxide (CO_2_) into a proton (H^+^) and bicarbonate (HCO_3_^−^)^1^^,^^2^^,^^6–11^. These proton export mechanisms, in concert with poor vascular drainage, are responsible to maintain an intracellular pH of 7.2–7.4, acidifying the extracellular pH to 6.2–6.8, which is strongly associated with the propagation, malignant progression, and resistance to chemotherapy and radiotherapy of tumours^1^^,^^2^^,^^6–11^. In detail, the CA IX and XII expression is strongly increased in many types of tumours^9^^,^^12–21^ and is downregulated by the wild-type von Hippel–Lindau tumour suppressor protein (pVHL)^2^^,^^22^^,^^23^. In some cancer cells, the *VHL* gene is mutated leading to the strong upregulation of tumour-associated CA isoforms as a consequence of constitutive HIF activation^24^^,^^25^. Recent studies have shown that isoform hCA IV, prevalently anchored on the membrane of the astrocytes, are responsible for regulating interstitial pH^26^ and for regulating transmembrane lactate transport, interacting with the chaperones of the monocarboxylate transporters in the brain cells^27–30^. Whereas targeting of hCA IV with inhibitors does not yet have clear antitumor therapeutic applications^31^, in the last decades, several studies corroborated CA IX and XII as targets for the development of carbonic anhydrases inhibitors (CAIs) as novel anticancer drugs and, to date, the sulphonamide inhibitor SLC-0111 is in phase two clinical trials as an antitumor agent^32–34^.

**Figure 1.**
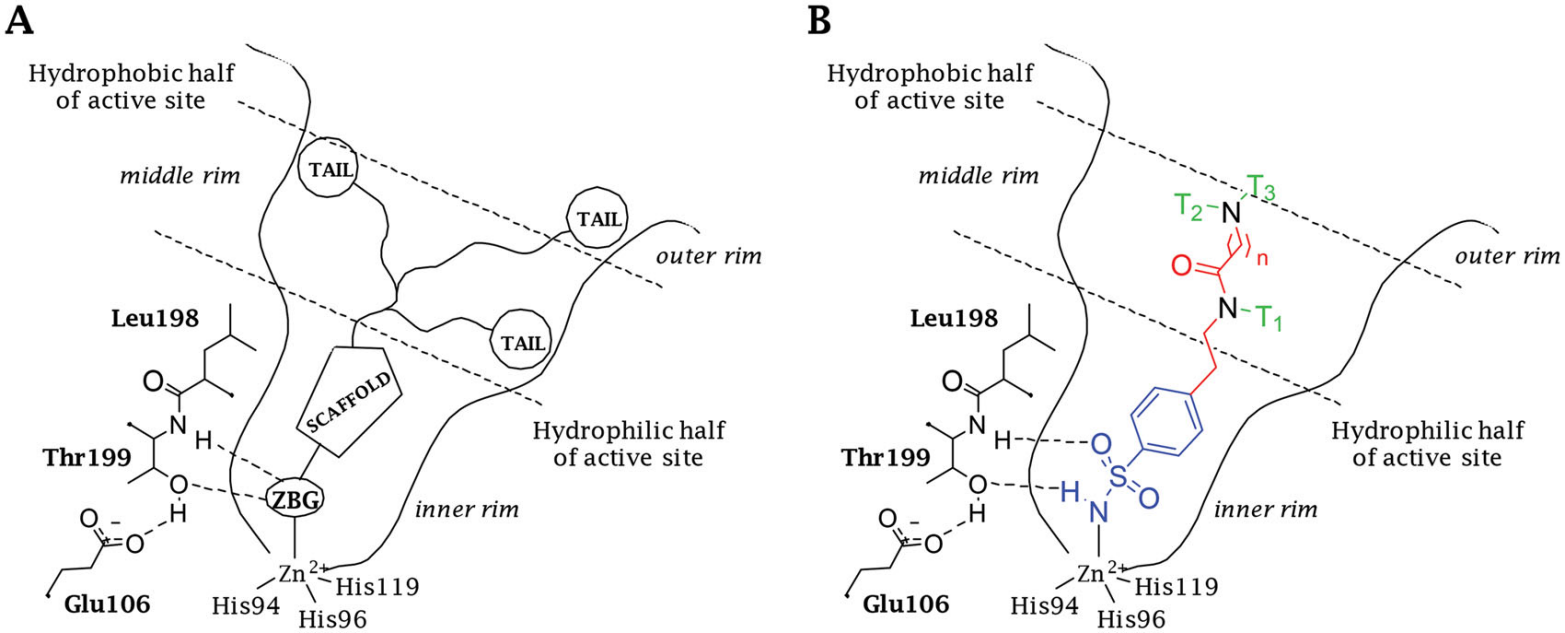
Schematic representation of the (A) “three-tails” approach for the design of zinc-binding CAIs. (B) Binding mode of a Three-Tailed Inhibitor (TTIs).

Among the large number of CAI chemotypes, the zinc binder sulphonamides led to many potent and fruitful inhibitory molecules^34^^,^^35^. However, their lack of selectivity and inability to discern among the 15 human (h) CA isoforms, prevents their wider use as therapeutic agents, at least for the first and second generation of such inhibitors^28^^,^^29^. In fact, the inhibition of ubiquitous and cytosolic isoforms hCA I and II is responsible for the side effect in the treatment with CAIs^34^^,^^35^. To overcome their promiscuous inhibition, the “three-tails approach” was applied as an extension of the previously proposed “tail” and “dual tails approach” (Figure 1(A))^36–39^. A careful 3D analysis has shown different dimensions of the 15 hCAs active site that together with diverse architecture and extension of the hydrophilic and lipophilic areas, line disparate pockets that could be targeted by specific tails^35^. The three-tails approach consists of appending three pendants of various nature on a CA inhibitory (CAI) scaffold (e.g. benzenesulfonamide) in order to interact with the most variable residues among the fifteen hCAs in the middle/outer rim of the active site, conferring to the sulphonamide inhibitors some important properties, such as water solubility^37^ and subsequently membrane (im)permeability^40^, improving the interactions with the hydrophilic and hydrophobic halves of the active sites and increasing the matching and fitting of the ligand-target contacts to attain the proper hCA selective inhibition^35^.

## 2. Material and methods

### 2.1. Chemistry

The synthesis and characterisation of sulphonamides **1–50** was reported earlier by our group^36^.

### 2.2. Carbonic anhydrase inhibition

An applied photophysics stopped-flow instrument has been used for assaying the CA catalysed CO_2_ hydration activity^41^ as reported earlier^42^. Enzyme concentrations were in the range 5–18 nM. All CA isoforms were recombinant ones obtained in-house as reported earlier^43^.

### 2.3. X-ray crystallography

#### 2.3.1. Protein expression and purification

Competent BL21 *Escherichia coli* cells were transformed with plasmid DNA containing the *hCA IX-mimic* gene using standard protocols as described earlier^44^^,^^45^.

#### 2.3.2. Crystallisation

Inhibitors were successfully cocrystalised with CAII and CAIX-mimic via the hanging-drop vapour diffusion method. 0.5 mL of mother liquor consisting of 1.6 M sodium citrate and 50 mM Tris at pH 7.8 was used in the wells for setting up crystal trays. Each cover slip contained a 1:1 ratio of 10 mg/mL protein to mother liquor. DMSO was used to dissolve inhibitors to 1 mM, with the drops final concentration ∼100 µM. Cocrystals of CAII and CAIX formed within a week.

#### 2.3.3. Data collection and processing

Diffraction data were collected *via* the F1 beamline at Cornell High Energy Synchrotron Source (CHESS, Ithaca, NY) at 0.977 Å wavelength and at Stanford Synchrotron Radiation Lightsource (SSRL, Menlo Park, CA). A Pilatus 6 M detector collected data sets with a crystal-to-detector distance of 270 mm, 1° oscillation, and 4 s image exposure, for a total of 180 images. Diffraction data were indexed and integrated with XDS^46^. Data were scaled in space group *P*2_1_ *via* AIMLESS^47^ from the CCP4 program suite^48^. Phases were determined *via* molecular replacement using PDB: 4ZAO^49^ as a search model. Modifications to the model, such as addition of inhibitor, ligand (glycerol), zinc, and water to the active site of CA were executed in Coot^50^ along with ligand PDB file modifications. Refinements were completed and ligand restraint files were created in Phenix^51^. Figures were generated with PyMol (Schrödinger). Protein-ligand bond lengths and active site interactions were observed with (LigPlot Plus, Hinxton, Cambridgeshire, UK)^52^ .

### 2.4. Antiproliferative assays

#### 2.4.1. Cell culture and treatments

Human prostate cancer cell line PC3, human breast cancer cell line ZR75-1, and human colon cancer cell line HT-29 were obtained from American Type Culture Collection (Rockville, MD). PC3, ZR75-1, and HT-29 were cultured in DMEM high glucose with 10% FBS in 5% CO_2_ atmosphere at 37 °C. Media contained 2 mM L-glutamine, 100 IU/mL penicillin, and 100 μg/mL streptomycin (Sigma, Milan, Italy). Cells were plated in 96-well cell culture (1.104/well) and, 24 h after, treated with the tested compounds (0 − 200 μM) for 48 h. Low oxygen conditions were acquired in a hypoxic workstation (Concept 400 anaerobic incubator, Ruskinn Technology Ltd., Bridgend, UK). The atmosphere in the chamber consisted of 1% O_2_ (hypoxia), 5% CO_2_, and residual N_2_. In parallel, normoxic (20% O_2_) dishes were incubated in air with 5% CO_2_.

#### 2.4.2. Cell viability assay

PC3, ZR75-1, and HT-29 cell viability were evaluated by the reduction of 3–(4,5-dimethylthiozol-2-yl)-2,5-diphenyltetrazolium bromide (MTT) as an index of mitochondrial compartment functionality. Cells were plated and treated as described. Post-treatment, after extensive washing, 1 mg/mL MTT was added into each well and incubated for 30 min at 37 °C. After washing, the formazan crystals were dissolved in 150 μL of DMSO. The absorbance was measured at 550 nm. Experiments were performed in quadruplicate on at least three different cell batches.

#### 2.4.3. Statistical analysis

Results were expressed as mean ± SEM, and the analysis of variance was performed by one-way ANOVA. A Bonferroni’s significant difference procedure was used as *post-hoc* comparison. *p* Values of less than .05 were considered as significant. Data were analysed using (Origin version 9.1 software, Hinxton, Cambridgeshire, UK).

## 3. Results and discussion

Several drug design studies of CAIs adopted the *p*-substituted benzenesulfonamide as a main scaffold against heteroaromatic sulphonamides to notably simplify the synthetic procedures and allow to focus on the attachment of variable pendants on the inhibitor structure^38^^,^^53^. A *p*-substituted benzenesulfonamide was adopted by us as a CAI scaffold to converge efforts and attention on studying the three-tailing effects on CA inhibition^36^. The structure of the three-tailed inhibitors (TTIs) was selected to merge an easy and versatile chemistry with the opportunity to expand it to many different chemical groups, which is significant for generating a range of tail combinations (Figure 1(B)). The synthetic strategies adopted to yield the TTI derivatives here discussed were previously reported by us^36^.

### 3.1. Carbonic anhydrase inhibition

In this first screening, mono-tailed (**1–7**) and three-tailed (**18–50**) compounds were analysed by a stopped-flow kinetic assay^41^ with: the tumour-associated isoforms CA IV, IX, and XII and the main off-target isoforms CA I and II, if considered the anticancer application (Table 1)^1^^,^^9–11^. The selectivity index of mono-tailed and three-tailed compounds *vs.* the off-target isoforms CA I and II are reported in Table S1 (Supplementary Information).

**Table 1.** Inhibition data of human CA isoforms I, II, IV, IX, and XII with sulphonamides **19–50** reported here and the standard sulphonamide inhibitor acetazolamide (AAZ) by a stopped-flow CO_2_ hydrase assay^41^.

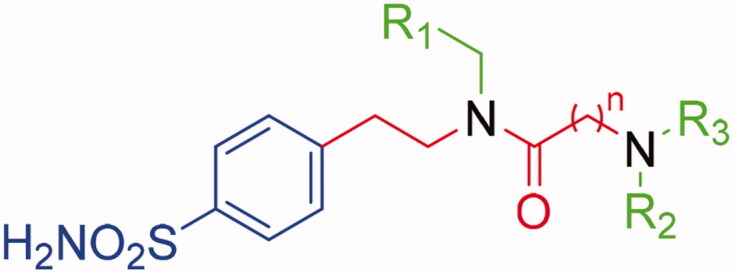									
Cmpd	*n*	R_1_	R_2_	R_3_	K_I_^a^				
					hCA I^b^	hCA II^b^	hCA IV^b^	hCA IX	hCA XII^b^
**1**	**–**	C_6_H_5_	**–**	**–**	95.3	98.4	2854.4	78.1	65.4
**2**	**–**	4-NO_2_-C_6_H_4_	**–**	**–**	224.3	120.9	1685.3	89.2	77.4
**3**	**–**	4-F-C_6_H_4_	**–**	**–**	112.8	78.5	1196.7	56.5	60.1
**4**	**–**	2-Naph	**–**	**–**	458.1	87.1	6248.1	71.2	78.6
**5**	**–**	Fu	**–**	**–**	68.4	62.8	1584.5	64.6	55.4
**6**	**–**	CH_2_CN	**–**	**–**	105.3	153.7	5547.2	104.7	113.2
**7**	**–**	CH_2_C_6_H_5_	**–**	**–**	278.4	89.1	3587.4	101.9	104.3
**18**	1	C_6_H_5_	CH_2_CH_3_	CH_2_CH_3_	786.6	8.3	4147.5	30.6	43.9
**19**	1	C_6_H_5_	CH_2_CH_3_	CH_2_C_6_H_5_	4210.4	391.6	>10,000	118.8	82.6
**20**	1	C_6_H_5_	CH_2_C_6_H_5_	CH_2_C_6_H_5_	865.9	412.3	>10,000	234.3	98.8
**21**	1	C_6_H_5_	(CH_2_)_4_CH_3_	(CH_2_)_4_CH_3_	506.1	124.5	>10,000	48.5	69.4
**22**	1	C_6_H_5_	(CH_2_)_5_CH_3_	(CH_2_)_5_CH_3_	878.7	237.0	>10,000	123.4	92.8
**23**	1	C_6_H_5_	(CH_2_)_7_CH_3_	(CH_2_)_7_CH_3_	946.7	843.8	>10,000	271.2	99.4
**24**	2	C_6_H_5_	CH_2_CH_3_	CH_2_CH_3_	184.7	8.9	3928.8	31.1	61.1
**25**	2	C_6_H_5_	CH_2_CH_3_	CH_2_C_6_H_5_	544.3	79.6	>10,000	165.7	90.4
**26**	2	C_6_H_5_	CH_2_C_6_H_5_	CH_2_C_6_H_5_	692.3	559.2	4640.8	106.4	302.5
**27**	2	C_6_H_5_	(CH_2_)_4_CH_3_	(CH_2_)_4_CH_3_	563.6	522.6	3244.8	133.8	100.3
**28**	2	C_6_H_5_	(CH_2_)_5_CH_3_	(CH_2_)_5_CH_3_	308.2	578.4	3455.4	94.2	77.8
**29**	2	C_6_H_5_	(CH_2_)_7_CH_3_	(CH_2_)_7_CH_3_	209.3	778.8	>10,000	146.5	280.0
**30**	2	CH_2_C_6_H_5_	(CH_2_)_5_CH_3_	(CH_2_)_5_CH_3_	518.4	780.8	3413.2	42.9	62.5
**31**	2	Fu	(CH_2_)_5_CH_3_	(CH_2_)_5_CH_3_	220.1	60.4	3153.7	47.1	9.7
**32**	1	2-Naph	(CH_2_)_5_CH_3_	(CH_2_)_5_CH_3_	541.4	4562.9	>10,000	295.5	61.7
**33**	1	CH_2_CN	(CH_2_)_5_CH_3_	(CH_2_)_5_CH_3_	395.9	52.5	3478.3	154.5	8.6
**34**	1	CH_2_C_6_H_5_	(CH_2_)_2_C_6_H_5_	(CH_2_)_2_CN	777.3	368.5	>10,000	32.3	75.5
**35**	1	Fu	(CH_2_)_2_C_6_H_5_	(CH_2_)_2_CN	300.8	73.2	457.4	95.1	8.7
**36**	1	4-F-C_6_H_5_	(CH_2_)_2_C_6_H_5_	(CH_2_)_2_CN	676.4	133.0	4133.8	3.0	9.8
**37**	1	2-Naph	(CH_2_)_2_C_6_H_5_	(CH_2_)_2_CN	685.0	247.5	3812.9	151.3	64.9
**38**	1	4-NO_2_-C_6_H_5_	(CH_2_)_2_C_6_H_5_	(CH_2_)_2_CN	407.5	264.2	2421.5	201.5	89.5
**39**	1	CH_2_CN	(CH_2_)_2_C_6_H_5_	(CH_2_)_2_CN	61.6	0.7	726.6	19.3	8.9
**40**	1	CH_2_C_6_H_5_	(CH_2_)_2_C_6_H_5_	(CH_2_)_3_NH_2_	242.4	367.3	2149.2	4.0	83.7
**41**	1	Fu	(CH_2_)_2_C_6_H_5_	(CH_2_)_3_NH_2_	246.7	57.0	374.1	11.1	42.7
**42**	1	4-F-C_6_H_5_	(CH_2_)_2_C_6_H_5_	(CH_2_)_3_NH_2_	451.4	30.4	365.3	4.8	0.6
**43**	1	2-Naph	(CH_2_)_2_C_6_H_5_	(CH_2_)_3_NH_2_	506.7	5.6	819.2	34.3	10.5
**44**	1	(CH_2_)_2_NH_2_	(CH_2_)_5_CH_3_	(CH_2_)_5_CH_3_	435.8	2924.8	913.9	115.6	32.5
**45**	1	CH_2_C_6_H_5_	(CH_2_)_2_C_6_H_5_	(CH_2_)_2_COOH	203.5	72.0	2330.5	7.5	29.7
**46**	1	Fu	(CH_2_)_2_C_6_H_5_	(CH_2_)_2_COOH	79.5	2.4	335.5	1.2	7.1
**47**	1	4-F-C_6_H_5_	(CH_2_)_2_C_6_H_5_	(CH_2_)_2_COOH	95.8	23.5	419.3	11.1	8.8
**48**	1	2-Naph	(CH_2_)_2_C_6_H_5_	(CH_2_)_2_COOH	197.0	72.5	680.6	22.1	6.8
**49**	1	CH_2_COOH	(CH_2_)_5_CH_3_	(CH_2_)_5_CH_3_	285.5	585.7	45.8	68.3	9.9
**50**	1	Fu	(CH_2_)_2_C_6_H_5_	(CH_2_)_2_CONHoleyl	737.9	132.0	1807.1	61.1	5.5
**AAZ**	–	–	–	–	250	12	74	25	5.7

^a^Mean from three different assays, by a stopped-flow technique (errors were in the range of ±5 − 10% of the reported values).

^b^Data reported in Bonardi et al. [36].

While the structure–activity relationship (SAR) against CA I, II, and IV have already been discussed in depth^36^, only the inhibitory action of mono-tailed inhibitors and TTIs against the tumour-associated isoforms CA IX and XII was here investigated and compared.

Generally, the inhibition data reported in Table 1 highlighted that mono-tailed compounds **1–7** were medium to high nanomolar inhibitors of CA I (*K_I_* = 68.4 − 458.1 nM), II (*K_I_* = 62.8 − 153.7 nM), IX (*K_I_* = 56.5 − 108.7 nM) and XII (*K_I_* = 55.4–113.2 nM), and weak inhibitors of CA IV with inhibition constant (*K_I_*) values in the low micromolar range (1.1 − 6.2 μM).

In detail, the tumour-associated isoforms CAs IX and XII were inhibited almost similarly by the single-tail compounds **1–7**. Nonetheless, derivatives **3** (*R*_1_= 4-F-C_6_H_4_) and **5** (*R*_1_= Fu) stood out as the best inhibitors of CA IX (*K_I_* = 56.5 nM) and XII (*K_I_* = 55.4 nM), respectively, whereas the cyanoalkyl- and phenethyl-tailed compounds **6** and **7** exhibit K_I_s above 100 nM against both isoforms.

As observed by the data in Table 1, the development of **1–7** upon inclusion of two other tails to synthesise compounds **18–50** significantly impacted the inhibition profiles against the panel of CA isoforms, as well as the selectivity indexes against CA I and II were improved (Table S1, Supplementary Information). In fact, TTIs showed lightly decreased or markedly improved inhibition of CA IX (*K_I_*s = 1.2 − 295.5 nM) and XII (*K_I_*s = 0.6 − 302.5 nM). CA IV remained the less inhibited isozyme, though inhibition improvement of one or two orders of magnitude was testified for some compounds (*K_I_*s = 45.8 –> 10,000 nM). On the whole, no significant improvement of the ubiquitous CA I inhibition was detected with TTIs (*K_I_*s = 79.5 − 4210.4 nM). The off-target CA II showed the inhibition profiles most affected, both positively and negatively, upon inclusion of additional tails on the scaffold of 1–7 (*K_I_*s = 0.7 − 4562.9 nM).

To better discuss TTIs’ SAR from Table 1, compounds and related data were distinguished in five subsets: (**i**) **18–29** (with R_1_ = C_6_H_5_), (ii) **30–33**, **44, 49** (with R_2_ = R_3_ = (CH_2_)_5_CH_3_), (iii) **34–39** (R_2_ = (CH_2_)_2_C_6_H_5_ and R_3_ = (CH_2_)_2_CN), (iv) **40–43** (R_2_ = (CH_2_)_2_C_6_H_5_ and R_3_ = (CH_2_)_3_NH_2_), and (v) **45–48** (R_2_ = (CH_2_)_2_C_6_H_5_ and R_3_ = (CH_2_)_2_COOH).

(i) Compounds **18**, **21**, and **24** resulted in the best CA IX inhibitors of the first subset (*K_I_* = 30.6, 48.5, and 31.1 nM, respectively) while **20** and **21** were the worst (*K_I_* = 234.3 and 271.2 nM). Instead, all derivatives potently inhibited the tumour-associated isoform CA XII with *K_I_* values below 100 nM, except for compounds **26** and **29** that were also the worst inhibitors among all the synthesised compounds against this isoform (*K_I_* = 280.0 and 302.5 nM), whereas **18** showed the best inhibitory profile of this series (*K_I_* = 43.9 nM). In this subset, compound **19** resulted in the fourth most selective CA XII inhibitor *vs.* CA I (CA I/CA XII = 51.0).

The importance of the linker length (*n* = 1, 2) is pointed out from the activity analysis of this first subset. In fact, the elongation of the chain between R_1_ and R_2_/R_3_ increased the activity against CA I, II, and IV which possess the smallest binding cavities, as a longer linker (*n* = 2) can shift the tails *R*_2_/*R*_3_ towards the rim of the active site removing the ligand-target steric encumbrance. On the other hand, the roomier active sites of CA IX and XII are able to host bulky substituents and the introduction of the linker *n* = 2, which drives out the tails R_2_/R_3_ from the active site, may decrease the activity weakening the ligand-target interactions.

(ii) Comparing the second subset (**30–33, 44, 49** with R_2_ = R_3_ = (CH_2_)_5_CH_3_) compounds with the first subset R_2_/R_3_-analogues **22** and **28**, it was highlighted that the introduction of CH_2_C_6_H_5_ and Fu in *R*_1_ increased the activity against both the tumour-associated isoforms, such as observed in compounds **30** (CA IX *K*_I_ = 42.9 nM; CA XII *K_I_* = 62.5 nM) and **31** (CA IX *K_I_* = 47.1 nM; CA XII *K_I_* = 9.7 nM), while the presence of 2-Naph (**32**) and CH_2_CN (**33**) increase the activity only against CA XII (*K_I_* = 61.7 and 8.6 nM, respectively). Furthermore, the tail R_1_ = CH_2_CN reduction of compounds **33** into amine **44** or its hydrolysis into carboxylic acid **49** worsened or did not affect the activity against CA XII (*K_I_* = 32.5 and 9.9 nM, respectively) but increased the inhibition profile *vs.* CA IX (*K*_I_ = 115.6 and 68.3 nM, respectively).

In particular, compounds **30**, **31**, and **49** were the best CA IX inhibitors of this second subset with K_I_ values below 100 nM (42.9, 47.1, and 68.3 nM, respectively), while derivatives **32**, **33**, and **44** were high nanomolar inhibitors of this isozyme (295.5, 154.5, and 115.6 nM, respectively). Compounds **44**, **30**, and **32** resulted, respectively, in the third (CA II/CA IX = 25.3), fourth (CA II/CA IX = 18.2), and fifth (CA II/CA IX = 15.4) most selective derivatives against CA IX with respect to the ubiquitous CA II.

In the case of CA XII all compounds showed a good activity: compounds **31** (*K_I_* = 9.7 nM), **33** (*K*_I_ = 8.6 nM), and **49** (*K_I_* = 9.9 nM) inhibited this isoform with K_I_ in the low nanomolar range while **30**, **32**, and **44** acted as medium nanomolar inhibitors (*K_I_* = 32.5–62.5 nM). TTIs **44**, **32**, and **49** resulted, respectively, in the first (CA II/CA XII = 90.0), second (CA II/CA XII = 74.0), and third (CA II/CA XII = 59.2) most selective compounds against CA XII *vs.* the off-target CA II.

Generally, it was observed for this subset that the concomitant presence of R_2_ = R_3_ = (CH_2_)_5_CH_3_ with substituent 2-Naph in R_1_ worsened the activity by 19 times against CA II (*K_I_* = 4.5 μM) but only 2.5 times against CA IX (*K_I_* = 295.5 nM) with respect to the analogue **22** (R_1_ = C_6_H_5_), increasing the CA II/CA IX selective index.

(iii) The third subset (**34–39**) is characterised by the introduction of a hydrophobic tail R_2_ = (CH_2_)_2_C_6_H_5_, a polar one R_3_ = (CH_2_)_2_CN, and a variable pendant *R*_1_. Only compounds **37** and **38** inhibited CA IX with *K_I_* values in the high nanomolar range (151.3 and 201.5 nM, respectively), derivatives **34**, **35**, and **39** were medium nanomolar inhibitors (*K_I_* = 32.3, 95.1, and 19.3 nM, respectively), while **36** (R_1_ = 4-F-C_6_H_5_) resulted in the first and second most selective inhibitor *vs.* the off-targets CA I (CA I/CA IX = 225.5) and CA II (CA II/CA IX = 44.3), respectively, among all the synthesised compounds with *K_I_* = 3.0 nM.

The target CA XII was strongly inhibited by all compounds of the subset with compounds **35**, **36**, and **39** acting in a low nanomolar range (*K_I_* = 8.7, 9.8, and 8.9 nM, respectively), while **34**, **37,** and **38** were medium nanomolar inhibitors (*K_I_* = 75.5, 64.9, and 89.5 nM, respectively). In this case, derivative **36** resulted also in the third most selective inhibitor against CA XII (CA I/CA XII = 69.8).

The comparison of compounds **37** and **39** from subset **iii** with the second subset analogues **32** and **33** (R_2_ = R_3_ = (CH_2_)_5_CH_3_) pointed out that the substitution of R_2_ and R_3_ with the tails (CH_2_)_2_C_6_H_5_ and (CH_2_)_2_CN, respectively, generally increased the activity against CA II, IV, and IX, with opposite effect against CA I and no significant effect against CA XII.

(iv) The fourth series (**40**–**43**) was obtained by reducing R_3_ = (CH_2_)_2_CN to amine tails in the aforesaid derivatives **34**–**37**, introducing a potentially positively charged pendant. This structural modification led to a general increment of the activity against CA I, II, IV, IX, and XII, suggesting that a strong polar interaction is favourable for the binding and might take place in all five active sites.

In detail, CA IX was potently inhibited in the low nanomolar range (*K_I_* = 4.0–34.3 nM) and inhibitors **42** (*K_I_* = 4.8 nM) and **40** (*K_I_* = 4.0 nM) resulted in the second and fourth most selective among all the synthesised compounds against this isozyme with respect to CA I (CA I/CA IX = 94.0 and 60.6, respectively), while derivatives **41** and **43** were medium nanomolar inhibitors with *K_I_* of 11.1 and 34.3 nM, respectively. Again, derivative **40** is the most selective inhibitor against CA IX *vs.* the off-target CA II (CA II/CA IX = 91.8).

The tumour-associated CA XII was strongly inhibited by **42** with a subnanomolar *K_I_* = 0.6 nM that makes it the most potent and selective compounds against this isoform (CA I/CA XII = 752.3; CA II/CA XII = 50.7), whereas **40** (*K_I_* = 83.7 nM), **41** (*K_I_* = 42.7 nM), and **43** (*K_I_* = 10.5 nM) acted with a *K_I_* in the medium nanomolar range.

(v) The fifth subset (**45**–**48**) obtained by the introduction of a potentially negatively charged tail in R_3_ showed a general increment of the inhibition activity against CA I, II, IV, IX, and XII compared to their analogues **34**, **35**, **37**, and **38**. Against CA IX compound **45** and **46** acted in the low nanomolar range (*K_I_* = 7.5 and 1.2 nM, respectively), where the last one resulted to be the most potent and the third most selective inhibitor *vs.* CA I (CA I/CA IX = 66), while **47** (*K_I_* = 11.1 nM) and **48** (*K_I_* = 22.1 nM) inhibited this isoform with *K_I_* in the medium nanomolar range.

Moreover, derivatives **46**–**48** were low nanomolar inhibitors of CA XII (*K_I_* = 7.1, 8.8, and 6.8 nM, respectively), whereas **48** and **46** resulted to be the second and third most potent inhibitors of this glaucoma-associated isoform, while compound **45** acted with a *K_I_* of 29.7 nM.

Comparing the fourth (**40**–**43**) and the fifth subset (**45**–**48**) it was detected that the presence of R_3_ = (CH_2_)_2_COOH in place of amine tails shifted the activity against CA I.

Finally, the loss of the hydrophilic tail R_3_ in **50** decreased the activity against CA I (*K_I_* = 737.9 nM), II (*K_I_* = 132.0 nM), IV (*K_I_* = 1.8 μM), and IX (*K_I_* = 61.1 nM) without effects against CA XII (*K_I_* = 5.5 nM), obtaining the second most potent and selective compound against this isoform (CA I/CA XII = 134.2).

### 3.2. X-ray crystallography

**Figure 2.**
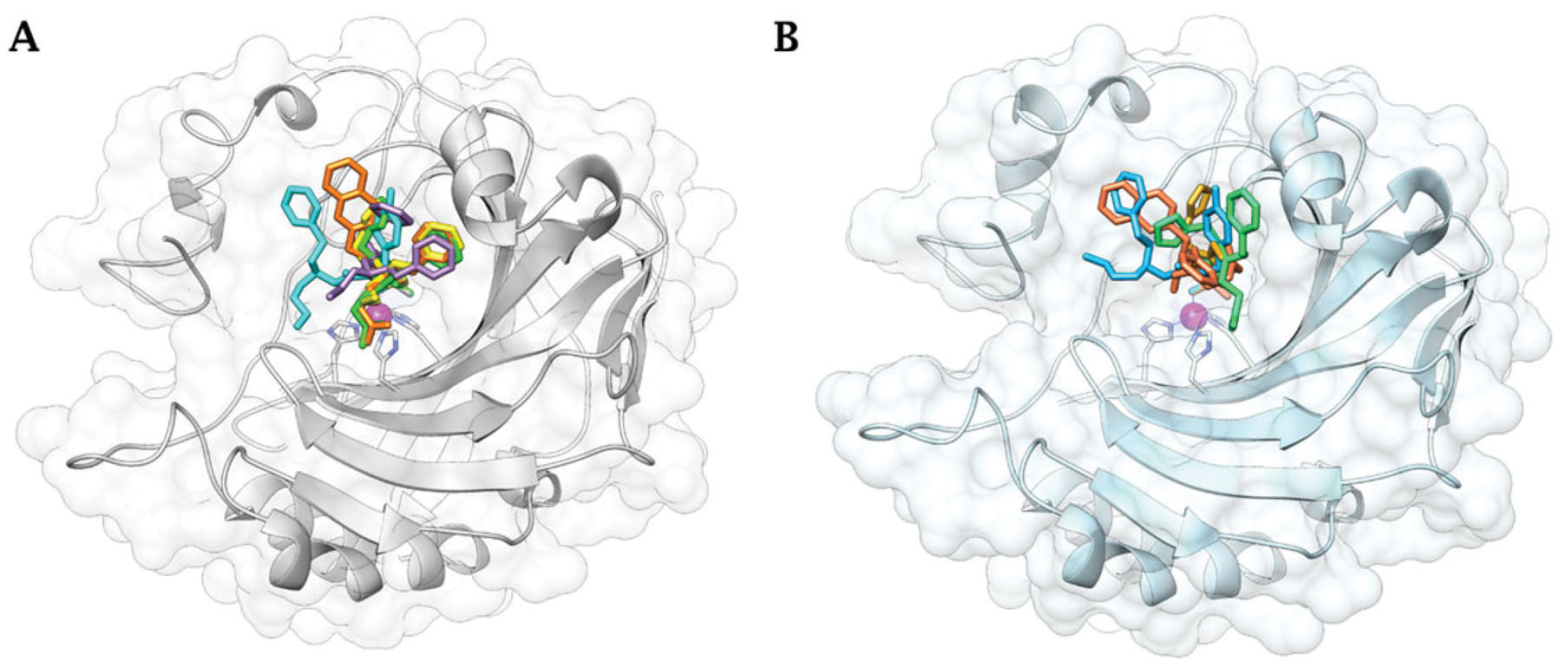
Cartoon representation of A) hCA II^36^ and B) hCA IX-mimic overlayed on a surface view with zinc (magenta sphere) and His94, His96, and His119 (sticks) shown in the active site with inhibitors bound. **34** (purple), **41** (yellow), **42** (cyan), **46** (green), and **48** (orange) are shown in both panels.

Co-crystallisation of hCA II^36^ and hCA IX-mimic with some of the new inhibitors resulted in solved crystal structures with resolutions between 1.39 and 1.56 Å (Figure 2 and Table S2, Supplementary Information). All inhibitors contain a conserved benzenesulfonamide in the same orientation that acts as a zinc-binding group that displaces zinc bound water (ZBW) to form a hydrogen bond between the amide backbone of Thr199 and oxygen of sulphonamide (2.9–3.0 Å). With these similarities in the inhibitors, any difference in observed binding affinity results from modifications to the tail regions.

**Figure 3.**
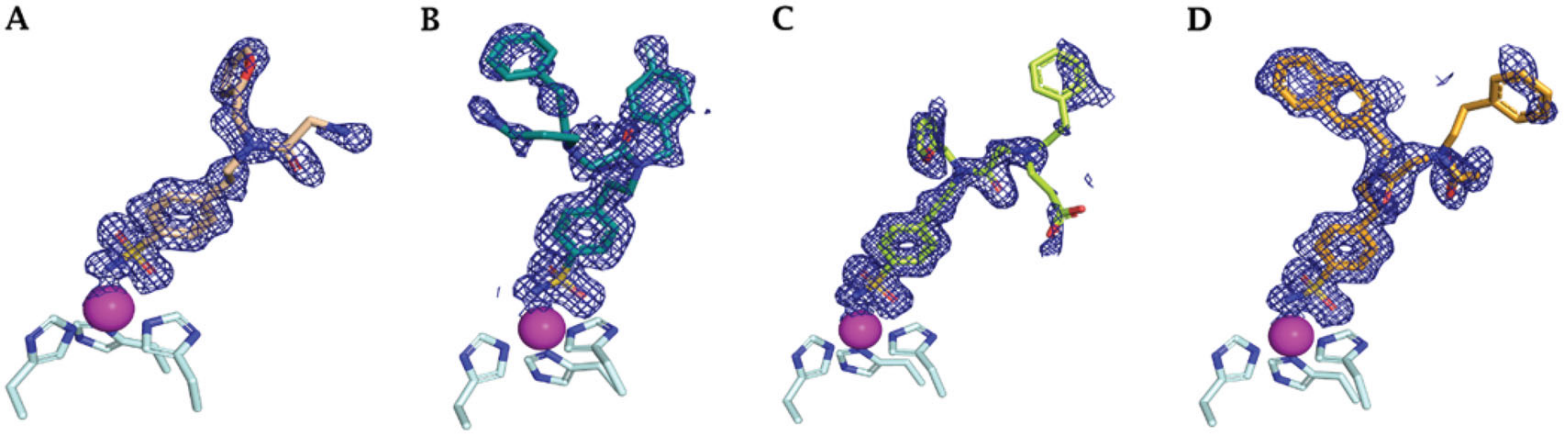
Electron density of A) **41**, B) **42**, C) **46**, and D) **48** in hCA IX-mimic active site with a sigma of 1.0.

**Figure 4.**
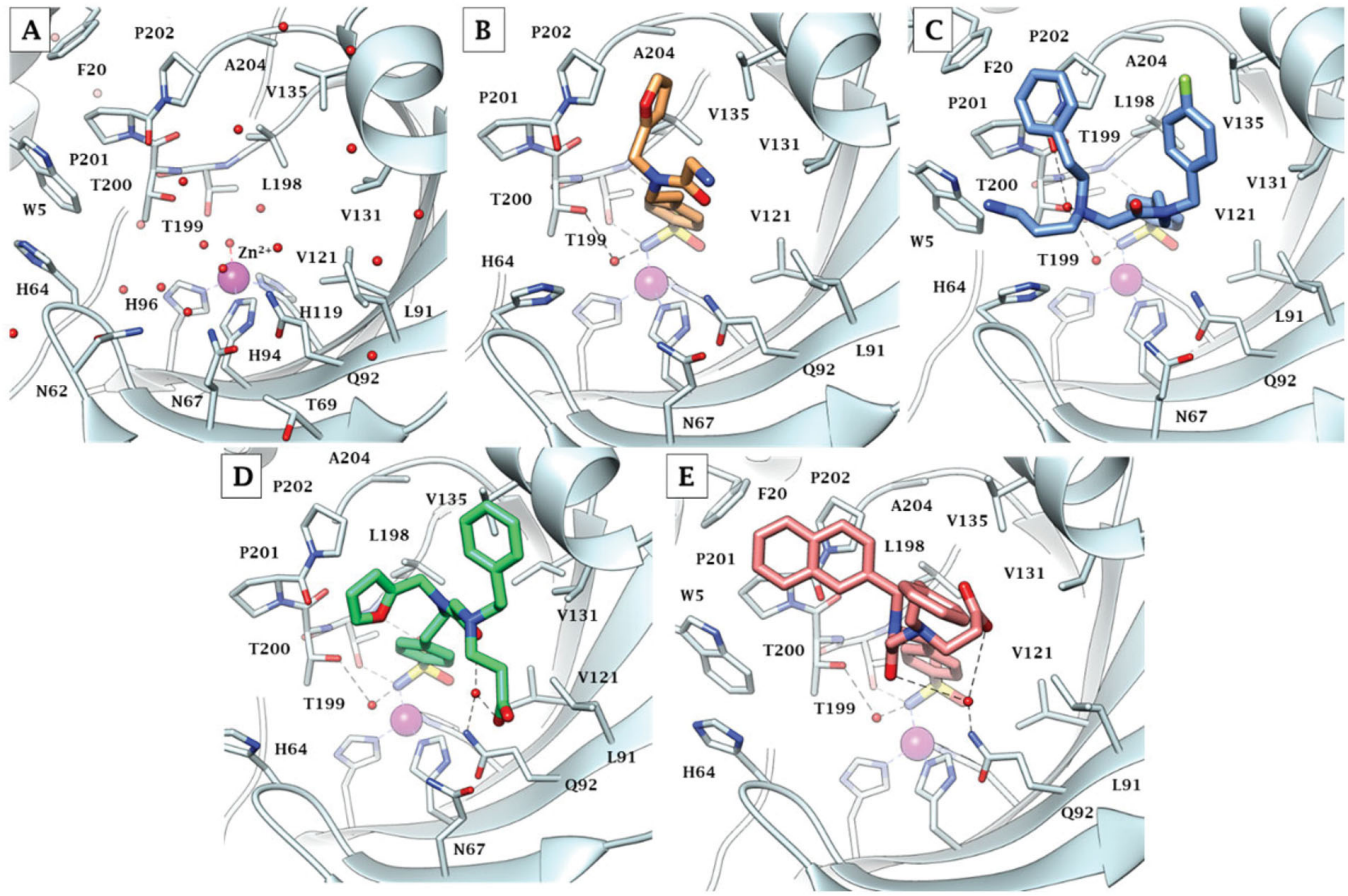
X-ray crystallography: active site view of hCA IX-mimic in adduct with A) no inhibitor, B) **41**, C) **42**, D) **46,** and E) **48**. H-bonds are depicted as black dashed lines. Water molecules involved in water-bridged H-bonds are shown as red spheres. Amino acids are labelled with one letter symbols: D, Asp; E, Glu; F, Phe; H, His; I, Ile; L, Leu; N, Asn; P, Pro; Q, Gln; T, Thr; V, Val; W, Trp.

In complex with hCA IX-mimic, compound **41** showed an observed omit map electron density lacking the whole entirety of T_2_ and T_3_ tails (PDB: 7SUW, Figure 3(A)). The T_1_ furyl moiety accommodated within the lipophilic pocket constituted by residues Val135, Leu198, Pro202, and Ala204, as it did within the hCA II active site (Figure 4(B)).

Compound **42** reported a strong observed omit map electron density, which may indicate stronger binding and/or a high binding occupancy (PDB: 7SUY, Figure 3(B)). Again, the swap from a furyl (**41**) to a 4-F-benzyl (**42**) in T_1_ induced the halo aromatic ring to lie over the lipophilic cleft made by Val131, Val135, Pro202, and Ala204. In contrast, the phenethyl portion in T_2_ interacts with the opposite side of the hCA IX-mimic hydrophobic half, namely with Trp5, Phe20, and Pro201 (Figure 4(C)). The protonated aminopropyl tail was exposed to bulk solvent.

Compound **46** had a weak observed omit map electron density around the benzene ring and carboxylic acid tail (PDB: 7SV8, Figure 3(C)). The furylmethyl tail flipped almost 180° with respect to that of derivative **41**, being in hydrophobic contact with Pro201 and Pro202. The phenethyl tail exhibited lipophilic interactions with Gly132 and Val135. The COO^-^ in T_3_ was involved in a water-mediated H-bond network with Gln92 that included the amide carbonyl group, while the T_3_ alkyl tail showed interactions with Leu91 (Figure 4(D)).

Compound **48** showed a weak observed omit map electron density around the benzene ring in T_2_ (PDB: 7SV1, Figure 3(D)). Expectedly, not being able to occupy the lipophilic pocket nearby Leu198 for steric hindrance reasons, the naphthyl ring in T_1_ lied over the region lined by Trp5, Phe20, Pro201, and Pro202. The benzene ring in T_3_ was interestingly found to interact with the outer portion of the *α*-helix including residues 130–136 and is partially exposed to bulk solvent, whereas the carboxyethyl tail folded back towards the cavity, making an intramolecular water bridge with the protonated amine group branching T_2_ and T_3_. However, it held water-bridged H-bonds to Gln92 together with the amide carbonyl group (Figure 4(E)).

Again, the binding mode exhibited by **46** was related to the best hCA IX inhibition measured *in vitro* (*K_I_* of 1.2 nM), though a minor difference was detected among the co-crystallised ligands with respect to hCA II. Likewise, the binding mode of **42**, that mostly deviated from that of the other ligands and also within hCA IX-mimic, led to an efficient hCA IX inhibition of 4.8 nM. The 20-fold drop of efficacy passing from **46** to **48** (K_I_ from 1.2 to 22.1 nM) might be related again to the furyl/napthyl switch that provoked a significant loss of favourable contacts within the active site.

**Figure 5.**
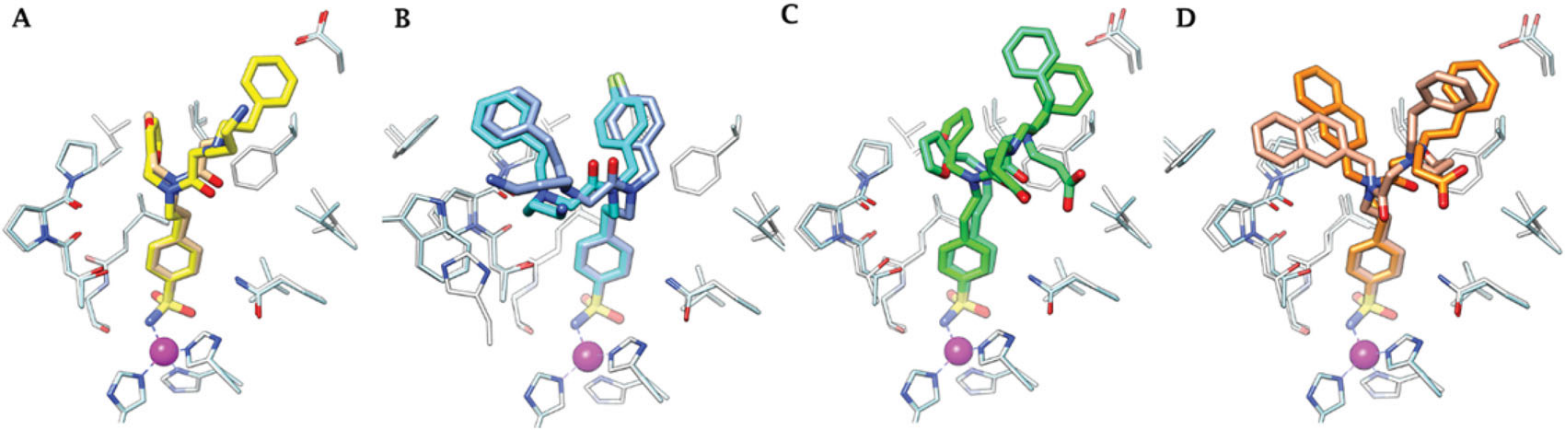
Superimposition of the crystallographic binding orientations adopted in the active site of hCA II (grey) [36] and hCA IX-mimic (light blue) for A) **41**, B) **42**, C) **46,** and D) **48**. Colours are as in Figure 6 of Bonardi et al. [36] and Figure 4.

Figure 5 depicts the superimposed binding orientations of compounds **41**, **42**, **46,** and **48** within the active site of hCA II^36^ and hCA IX-mimic. Although partially missing electron density was observed for compound **41** in the hCA IX-mimic active site, a similar orientation to the ligand binding mode in hCA II was observed for T_1_ and for the linker up to the T_2_/T_3_ branching junction. Hence, very similar ligand orientations exist for inhibitors **41** (Figure 5(A)) and **42** (Figure 5(B)) bound to hCA II and hCA IX-mimic. In contrary, greater differences were detected when ligands **46** and **48** bound to the active site of the two isoforms. Nonetheless, it should be stressed that all four compounds showed a significantly greater efficacy as inhibitors of hCA IX than hCA II, with selectivity that spans between 2- and 6-fold. In fact, hCA II had an average K_I_ of 41 nM (for compounds **41**, **42**, **46**, and **48**), whereas hCA IX had an average *K_I_* of 10 nM for these same inhibitors. In this context, Val131 in hCA IX compared to Phe131 in hCA II is the major difference in active sites between the two isoforms. Inhibitors more easily enter the active site in hCA IX due to the smaller amino acid at residue 131. What is more, Phe131 of hCA II can produce less favourable positioning and conformational geometry of the inhibitors with respect to hCA IX-mimic.

**Figure 6.**
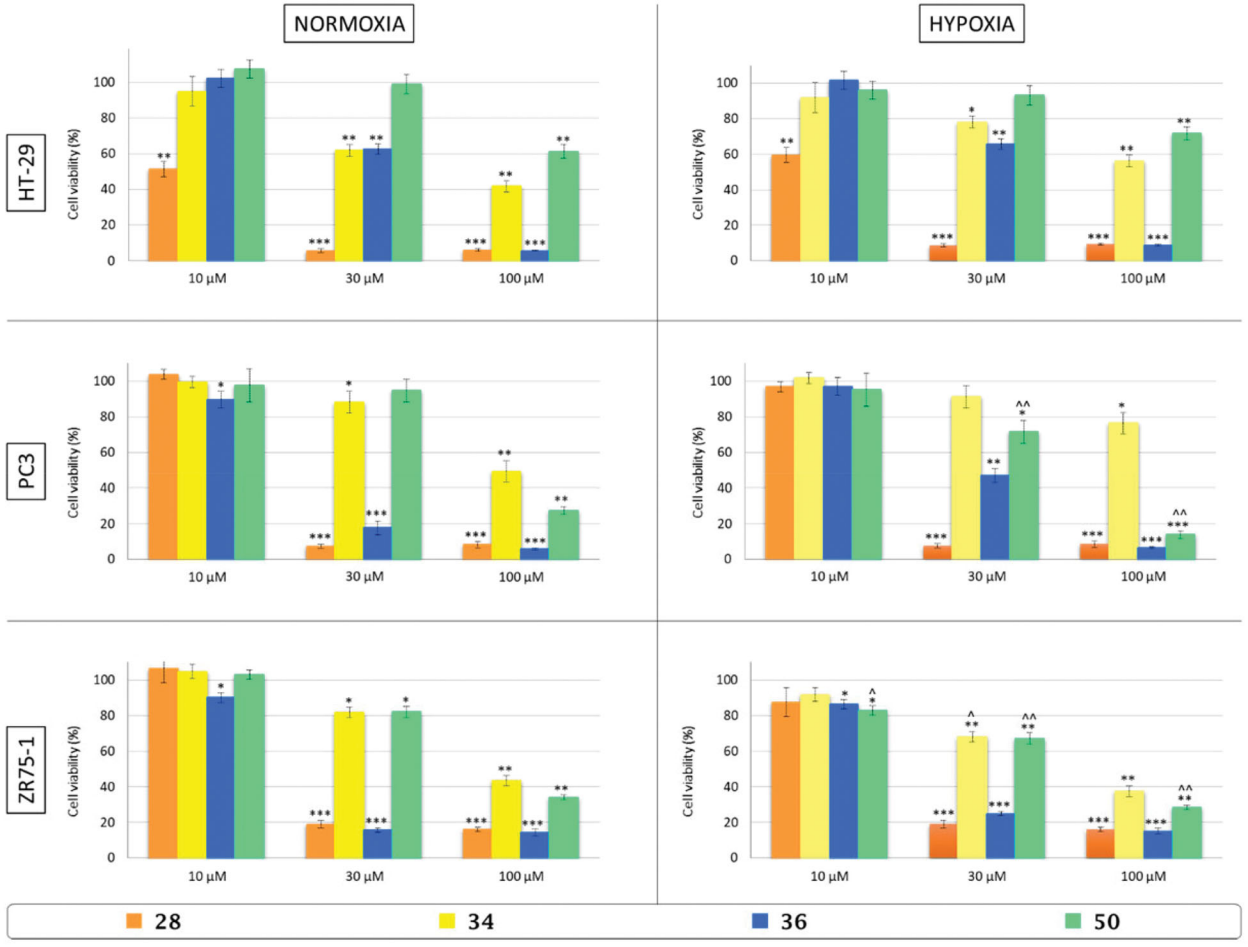
*In vitro* cell viability assay of colon adenocarcinoma (HT-29), prostate adenocarcinoma (PC3), and breast cancer (ZR75-1) cell lines after 48 h of treatment with three different concentrations (10, 30, and 100 µM) of three-tailed inhibitors **28** (orange), **34** (yellow), **36** (blue), and **50** (green) in normoxic (21% O_2_) and hypoxic (3% O_2_) conditions. Control cells are arbitrarly set at 100% and results are expressed as the mean ± SEM of three experiments. One-way ANOVA was performed followed by a Bonferroni’s significant difference procedure. *p < .05, **p < .01, and ***p < .001 *vs.* control; ^∧^*p* < .05 and ^∧∧^*p* < .01 *vs.* normoxia.

Comparing compound **46** between hCA II and hCA IX-mimic shows that in the latter, the amide linker shifted towards the hydrophobic region most likely as a result of less steric hindrance from Val131. As a result, the two cyclic tails rotate in towards the active site, preventing clashes with the enzymes surface residues (Figure 5(C)). As for compound **48**, there was also less steric hindrance from Val131 which allowed the phenyl tails to rotate about the linker (Figure 5(D)). The clearer case was that of compound **42**; although a very similar orientation was observed when bound within the two active sites, the inhibitor tails slightly moved towards the hydrophobic half of hCA IX-mimic accordingly to the less steric hindrance from Val131 (Figure 5(B)) just enough to improve the binding and thus inhibition efficacy.

### 3.3. Antiproliferative studies

Hypoxic tumours are a heterogeneous mass of cells with different degrees of oxygen supply^1^^,^^6–8^^,^^54^. The cells in the internal part of the mass grow under hypoxic conditions while the external ones have a more physiological supply of oxygen. In normoxic cells (where CA IX and XII are normally expressed on the membranes), CAIs could predominantly act on the cytosolic isoforms, blocking the ability of cells to maintain the intracellular and extracellular pH values compliant for their survival. Instead, in the hypoxic ones, CAIs also inhibit the tumour-membrane-associated isoforms of CA IX and XII that result overexpressed in these conditions. Obviously, achieving selective inhibition of CA IX and XII, expressed in both cell types, is preferable to avoid the side effects related to the inhibition of the off-target isoform also present in the healthy cells with CAIs.

To evaluate *in vitro* the effects on the viability of colon (HT29), prostate adenocarcinoma (PC3) and breast cancer (ZR75-1) cell lines, TTIs **28**, **34**, **36**, and **50** were selected among all the synthesised derivatives for their selectivity against CA IX and XII and also considering the nature of the tails. Cells were incubated for 48 h with three different concentration of inhibitors (10, 30, and 100 μM) in normoxic (21% O_2_) and hypoxic (3% O_2_) conditions.

All compounds act in a dose-dependent manner similarly in normoxia and hypoxia against HT29 and PC3 cancer cell lines, resulting in a possession of a slightly most potent effect in hypoxic conditions *vs.* the cell line ZR75-1. Moreover, inhibitors **28** and **36** were the most active against all the used cell lines while derivative **50** reduced the ZR75-1 cell viability.

In detail, inhibitor **28** was effective at the lowest concentration (10 µM) only against HT-29 cells, decreasing viability by about 55% in normoxia and 40% in hypoxia. The other concentrations (30–100 µM) further reduced viability by more than 90% in HT-29 and PC3 cell lines and were slightly less effective in ZR75-1 where the reduction was around 80%.

Inhibitors **34**, **36,** and **50**, on the other hand, have no effect at a concentration of 10 µM, except for compound **36** on PC3 cells in normoxia and on ZR75-1 cells in both conditions (15% of decrease). Interestingly, compound **50** at 10 µM on ZR75-1 was effective in hypoxic condition compared to normoxia, inducing a 15% of reduction (Figure 6).

Inhibitor **34** at a concentration of 30 µM affected the viability under normoxic condition up to 40% in HT-29 cells and 15–20% in PC3 and ZR75-1 cells. In hypoxic condition, it was less effective on HT-29 and PC3 cell lines, while ZR75-1 cell viability decreased by 30% compared to control and by10–15% respect to normoxic condition. Even the concentration 100 µM of inhibitor **34** was able to induce cell death in a similar percentage for the three cell lines (50–60%) under normoxic condition, and in hypoxia ZR75-1 cells were confirmed as the most sensitive compared to the other cell lines.

Derivative **36** was more effective against PC3 and ZR75-1 at 30 µM and 100 µM in both conditions, with a reduction of cell viability by around 80–90%, except for the 30 µM in hypoxia on PC3 cells where the viability decreased only by 50%. On HT-29 cells inhibitor **36** induced a 40% and 90% reduction at 30 and 100 µM, respectively, in both conditions.

Finally, inhibitor **50** had no effect at 30 µM on HT-29 cells, and at 100 µM a 40% reduction was observed in both conditions; on PC3 cells under hypoxic condition the concentration 30 µM of derivative **50** induced a reduction by 30%, while at 100 µM a more effective action under hypoxic condition was determined (almost 50% compared to normoxia); also ZR75-1 cells were sensitive to inhibitor **50,** which was active at 30 µM (respectively, 20% and 30% of reduction in normoxia and hypoxia) and at 100 µM was able to induce 65% of death in normoxia and a significant further decrease in hypoxia (5–10% less)^55–58^.

## 4. Conclusion

In recent decades the human (h) CAs (EC 4.2.1.1) isoforms IX and XII were validated as anticancer targets against solid hypoxic tumours. The lack of selectivity of sulphonamide CAIs prevents their wider use as therapeutic agents owing to the side effects onset, mainly due to the inhibition of the ubiquitous human (h) CA I and II. To overcome this issue the “three-tails approach” is here proposed as an extension of the forerunner “tail” and “dual-tail approach” to fully exploit the amino acid differences at the medium/outer active sites rim among the different hCA active sites. The majority of the thirty-three synthesised TTIs resulted in a higher selectivity against the tumour-associated isoforms hCA IX and XII with respect to the off-targets hCA I and II than the mono-tailed compounds (CA I/CA IX = 1.8–225.5; CA II/CA IX = 1.3–91.8; CA I/CA XII = 2.3–752.3; CA II/CA XII = 1.3–90.0). X-ray crystallography studies were performed to investigate the binding mode of four TTIs (**41**, **42**, **46**, and **48**) in complex with hCA IX mimic. Moreover, the ability of the most potent and selective TTIs (**28**, **34**, **36**, and **50**) to reduce *in vitro* the viability of colon (HT-29), prostate adenocarcinoma (PC3), and breast cancer (ZR75-1) cell lines was evaluated in normoxic (21% O_2_) and hypoxic (3% O_2_) conditions. In particular, all tested compounds act in a concentration-dependent manner similarly in normoxia and hypoxia against HT-29 and PC3 cancer cell lines, and with a slightly most potent effect in hypoxic conditions against ZR75-1 cell line. Moreover, inhibitors **28** and **36** resulted in the most active derivatives against all the cell lines used while derivative **50** was able to strongly affect PC3 and ZR75-1 cell viability under hypoxic condition compared to normoxia.

## Supplementary Material

Supplemental Material
