# Supplementary material for: The three-tails approach as a new strategy to improve selectivity of action of sulphonamide inhibitors against tumour-associated carbonic anhydrase IX and XII
Source: J Enzyme Inhib Med Chem. 2022 Mar 21;37(1):930–9. doi: 10.1080/14756366.2022.2053526 (PMC8942523; doi:10.1080/14756366.2022.2053526)
Supplement: Supplemental Material [file IENZ_A_2053526_SM9927.pdf]

Supplementary Information

The three-tails approach as a new strategy to improve selectivity of action of sulfonamide inhibitors against tumor-associated carbonic anhydrase IX and XII.

Alessandro Bonardi, Silvia Bua, Jacob Combs, Carrie Lomelino, Jacob Andring, Sameh Mohamed Osman, Alessandra Toti, Lorenzo Di Cesare Mannelli, Paola Gratteri, Carla Ghelardini, Robert McKenna, Alessio Nocentini,* Claudiu T. Supuran*

Selectivity Index (*SI*) of Carbonic Anhydrase Inhibition S2

X-ray crystallography data S3

Table S1. Selective index (*SI*) for the single-tail compounds **1-7** and **TTIs 18-50** calculated as ratio between $K_{I\ CA\ I}$ and $K_{I\ CA\ II}$

Cmpd	R_1	R_2	R_3	*SI*					
				**hCA I/IV**	**hCA II/IV**	**hCA I/IX**	**hCA II/IX**	**hCA I/XII**	**hCA II/XII**
**1**	C_6H_5			0.03	0.03	1.2	1.3	1.5	1.5
**2**	4-NO_2-C_6H_4	-	-	0.1	0.07	2.5	1.4	2.9	1.6
**3**	4-F-C_6H_4	-	-	0.09	0.06	2.0	1.4	1.9	1.3
**4**	2-Naph	-	-	0.07	0.01	6.4	1.2	5.8	1.1
**5**	Fu	-	-	0.04	0.04	1.1	1.0	1.2	1.1
**6**	CH_2CN	-	-	0.02	0.03	1.0	1.5	0.9	1.4
**7**	$CH_2C_6H_5$	-	-	0.08	0.02	2.7	0.9	2.7	0.9
**18**	C_6H_5	CH_2CH_3	CH_2CH_3	0.2	0.002	25.7	0.3	17.9	0.2
**19**	C_6H_5	CH_2CH_3	$CH_2C_6H_5$	<0.4	<0.04	35.4	3.3	51.0	4.7
**20**	C_6H_5	$CH_2C_6H_5$	$CH_2C_6H_5$	<0.09	<0.04	3.7	1.8	3.7	8.8
**21**	C_6H_5	$(CH_2)_4CH_3$	$(CH_2)_4CH_3$	<0.05	<0.01	10.4	2.6	7.3	1.8
**22**	C_6H_5	$(CH_2)_5CH_3$	$(CH_2)_5CH_3$	<0.09	<0.02	7.1	1.9	2.6	9.5
**23**	C_6H_5	$(CH_2)_7CH_3$	$(CH_2)_7CH_3$	<0.09	<0.08	4.3	3.1	9.5	8.5
**24**	C_6H_5	CH_2CH_3	CH_2CH_3	0.04	0.002	5.9	0.3	3.0	0.1
**25**	C_6H_5	CH_2CH_3	$CH_2C_6H_5$	<0.05	<0.008	3.3	0.5	6.0	0.9
**26**	C_6H_5	$CH_2C_6H_5$	$CH_2C_6H_5$	0.2	0.1	6.5	5.2	2.3	1.8
**27**	C_6H_5	$(CH_2)_4CH_3$	$(CH_2)_4CH_3$	0.2	0.2	4.2	3.9	5.6	5.2
**28**	C_6H_5	$(CH_2)_5CH_3$	$(CH_2)_5CH_3$	0.09	0.2	3.3	6.1	4.0	7.4
**29**	C_6H_5	$(CH_2)_7CH_3$	$(CH_2)_7CH_3$	<0.02	<0.08	1.4	5.3	0.7	2.8
**30**	$CH_2C_6H_5$	$(CH_2)_5CH_3$	$(CH_2)_5CH_3$	0.2	0.2	12.1	18.2	8.3	12.5
**31**	Fu	$(CH_2)_5CH_3$	$(CH_2)_5CH_3$	0.07	0.02	4.7	1.3	22.7	6.2
**32**	2-Naph	$(CH_2)_5CH_3$	$(CH_2)_5CH_3$	<0.05	<0.5	1.8	15.4	8.8	74.0
**33**	CH_2CN	$(CH_2)_5CH_3$	$(CH_2)_5CH_3$	0.1	0.02	2.6	0.3	46.0	6.1
**34**	$CH_2C_6H_5$	$(CH_2)_2C_6H_5$	$(CH_2)_2CN$	<0.08	<0.04	24.1	11.4	10.3	4.9
**35**	Fu	$(CH_2)_2C_6H_5$	$(CH_2)_2CN$	0.7	0.2	3.2	0.8	34.6	8.4
**36**	4-F-C_6H_4	$(CH_2)_2C_6H_5$	$(CH_2)_2CN$	0.2	0.03	225.5	44.3	69.8	13.6
**37**	2-Naph	$(CH_2)_2C_6H_5$	$(CH_2)_2CN$	0.2	0.06	4.5	1.6	10.5	3.8
**38**	4-NO_2-C_6H_4	$(CH_2)_2C_6H_5$	$(CH_2)_2CN$	0.2	0.1	2.0	1.3	4.6	3.0
**39**	CH_2CN	$(CH_2)_2C_6H_5$	$(CH_2)_2CN$	0.08	0.001	3.2	0.03	6.9	0.08
**40**	$CH_2C_6H_5$	$(CH_2)_2C_6H_5$	$(CH_2)_3NH_2$	0.1	0.2	60.6	91.8	2.9	4.4
**41**	Fu	$(CH_2)_2C_6H_5$	$(CH_2)_3NH_2$	0.7	0.2	22.2	5.1	5.8	1.3
**42**	4-F-C_6H_4	$(CH_2)_2C_6H_5$	$(CH_2)_3NH_2$	1.2	0.08	94.0	6.3	752.3	50.7
**43**	2-Naph	$(CH_2)_2C_6H_5$	$(CH_2)_3NH_2$	0.6	0.007	14.8	0.2	48.3	0.5
**44**	$(CH_2)_2NH_2$	$(CH_2)_5CH_3$	$(CH_2)_5CH_3$	0.5	3.2	3.8	25.3	13.4	90.0
**45**	$CH_2C_6H_5$	$(CH_2)_2C_6H_5$	$(CH_2)_2COOH$	0.09	0.03	27.1	9.6	6.9	2.4
**46**	Fu	$(CH_2)_2C_6H_5$	$(CH_2)_2COOH$	0.2	0.007	66.3	2.0	11.2	0.3
**47**	4-F-C_6H_4	$(CH_2)_2C_6H_5$	$(CH_2)_2COOH$	0.2	0.06	8.6	2.1	10.9	2.7
**48**	2-Naph	$(CH_2)_2C_6H_5$	$(CH_2)_2COOH$	0.3	0.1	8.9	3.3	29.0	10.7
**49**	CH_2COOH	$(CH_2)_5CH_3$	$(CH_2)_5CH_3$	6.2	12.8	4.1	8.6	28.8	59.2
**50**	Fu	$(CH_2)_2C_6H_5$	$(CH_2)_2$CONHoleyl	0.4	0.07	12.1	2.2	134.2	24.0

Table S2. X-ray crystallography data collection and refinement statistics on inhibitor bound CA IX-mimic crystal structures.

**Inhibitor**	**41**	**42**	**46**	**48**
**Space group**	$P2_1$	$P2_1$	$P2_1$	$P2_1$
**Cell dimension**	42.0, 41.5	41.9, 41.4	42.1, 41.7	41.8, 41.6
**a, b, c, β (Å, deg)**	71.7, 103.8	72.0, 103.8	71.9, 103.9	72.1, 103.8
**Resolution (Å)**	20.95 – 1.41	24.48 – 1.56	22.7 – 1.46	35.02 – 1.39
**Highest resolution shell (Å)**	(1.46 – 1.41)	(1.62 – 1.56)	(1.51 – 1.46)	(1.44 – 1.39)
**Total reflections**	13815	10549	13124	10300
$I/\sigma(I)$	18.6 (2.4)	15.7 (2.2)	19.8 (2.7)	21.1 (3.2)
**Redundancy**	3.3 (3.1)	3.4 (3.2)	3.4 (3.4)	3.3 (2.8)
**Completeness (%)**	98.6 (95.1)	97.1 (94.7)	95.1 (90.9)	94.9 (75.8)
R_{sym} [a]	4.12 (47.6)	4.49 (48.4)	3.51 (41.8)	2.93 (25.2)
R_{crys} [b]	16.5 (24.4)	17.1 (22.8)	16.14 (22.7)	15.4 (20.2)
R_{free} [c]	18.7 (27.1)	19.8 (26.9)	19.0 (26.8)	17.7 (22.9)
R_{pim} [d]	2.65 (31.4)	2.88 (31.3)	2.23 (26.3)	1.88 (17.9)
**# of atoms:**				
**protein**	2054	2048	2080	2071
**ligand**	32	46	45	50
**water**	198	154	185	182
**Protein residues**	257	257	257	257
**Ramachandran stats (%):**				
**favored**	96.1	96.9	96.1	96.5
**allowed**	3.9	3.1	3.9	3.5
**Avg. B-factors (Å^2):**				
**main-chain**	16.7	19.9	21.1	19.2
**side-chain**	17.5	20.6	22.0	20.2
**inhibitor**	26.4	36.0	36.4	36.6
**solvent**	24.5	25.2	27.8	27.5
**RMSD for:**				
**bond lengths (Å)**	0.01	0.011	0.009	0.018
**angles (deg)**	1.22	1.1	1.06	1.19

[a] $R_{sym} = (\Sigma|I - \langle I\rangle|/\Sigma \langle I\rangle) \times 100$. [b] $R_{cryst} = (\Sigma|F_o - F_c|/\Sigma|F_o|) \times 100$. [c] R_{free} is calculated in the same way as R_{cryst} except it is for data omitted from refinement (5% of reflections for all data sets). [d] $R_{pim} = [(\Sigma\sqrt{1/N - 1})\Sigma|I - \langle I\rangle|/\Sigma \langle I\rangle] \times 100$. [e] Values in parentheses correspond to those of the highest resolution shell.
